# Relationship Between Sleep Apnea and Coronary Artery Calcium in Patients With Ischemic Stroke

**DOI:** 10.3389/fneur.2019.00819

**Published:** 2019-07-31

**Authors:** Kyoo Ho Cho, Dong Hyun Lee, Kyung Min Kim, Yun Ho Choi, Hyo Suk Nam, Ji Hoe Heo, Kyoung Heo, Young Dae Kim

**Affiliations:** ^1^Department of Neurology, Yonsei University College of Medicine, Seoul, South Korea; ^2^Department of Neurology, Incheon St. Mary's Hospital, College of Medicine, The Catholic University of Korea, Incheon, South Korea

**Keywords:** stroke, coronary artery disease, coronary computed tomography, sleep apnea, polysomnography

## Abstract

**Study Objectives:** Coronary artery disease is considered to be the major cause of death amongst patients with ischemic stroke. The coronary artery calcium (CAC) score is related not only to sleep-disordered breathing, but also with future risk of cardiovascular mortality. We investigated the association between the severity of sleep-disordered breathing and CAC score in patients with ischemic stroke.

**Methods:** We included 32 patients who underwent coronary multichannel computed tomography and polysomnography (within 2 years of the stroke event) amongst the patients admitted to our clinic due to acute ischemic stroke. We investigated vascular risk factors, polysomnography findings, and sleep questionnaire scores, and their relationships with the CAC score.

**Results:** All patients were found to have sleep apnea of any degree, and 23 (72%) had severe sleep apnea. Twenty-three (72%) patients had a positive CAC score. Higher CAC scores were associated with elevated respiratory disturbance index (RDI), apnea index, oxygen desaturation index, and STOP-BANG test scores. Multivariate analysis after adjusting for potential confounding factors revealed independent relationships between the CAC score and the RDI (ß [SE] = 5.3 [2.1], *p* = 0.01), oxygen desaturation index (ß [SE] = 6.8 [2.8], *p* = 0.02), and STOP-BANG test score (ß [SE] = 90.3 [37.7], *p* = 0.02).

**Conclusion:** Our findings indicate a relationship between coronary atherosclerotic burden measured by the CAC score and the severity of sleep apnea. Performing polysomnography could be useful for investigating the severity of hidden coronary artery disease among these patients.

**Brief Summary:**

**Current Knowledge/Study Rationale:** The effect of sleep apnea on coronary artery disease in patients with ischemic stroke has not been explored. We investigated the relationship between sleep apnea, its related characteristics and the coronary artery calcium score in patients with stroke.

**Study Impact:** Our findings reveal a close relationship between the atherosclerosis-related burden measured by the coronary artery calcium score and the severity of sleep apnea that persisted after adjusting for confounding variables related to the risk of coronary artery disease. Proper detection and treatment of sleep apnea might mitigate the risk of future coronary events in patients with ischemic stroke.

## Introduction

Sleep apnea is a condition characterized by recurrent episodes of airflow limitation during sleep resulting in frequent awakening, hypoxia, and sympathetic activation (1). Sleep apnea can lead to a decrease in the quality of life by causing impairments in daytime alertness and cognitive function. Furthermore, individuals with sleep apnea have been reported to show an increased risk of cardiovascular diseases (2, 3).

Coronary artery occlusive disease (CAOD) is common in patients with stroke (4). Previous studies have shown that 30–70% patients with stroke are diagnosed with CAOD, which is associated with long-term cardiovascular outcomes even in patients without any definite clinical history of CAOD (5, 6). Computed tomography-based coronary artery calcium (CAC) score, a non-invasive and simple quantitative measure of atherosclerotic burden in the coronary artery, can be used for the prediction of occult coronary artery disease as a surrogate marker for future cardiovascular events (7, 8). Given the association between the risk of CAOD and prevalence of sleep apnea in an otherwise healthy population (9), the atherosclerotic burden on the coronary artery may be dependent on the severity of sleep apnea in patients with stroke in whom sleep apnea is common. However, this association has not been duly explored until now.

In the present study, we investigated the association between the severity of sleep apnea, and the CAC score in patients with ischemic stroke with no previous history of CAD.

## Materials and Methods

### Study Population

This study was a retrospective observational study conducted to investigate the relationship between atherosclerotic burden and sleep disorder in patients with ischemic stroke or transient ischemic attack (TIA). Cerebral infarction was confirmed with brain imaging in patients with sudden and focal neurologic deficits of a presumed vascular etiology. During hospitalization, all the patients had undergone brain imaging studies, cerebral angiographic studies, 12-lead electrocardiography, and standard blood tests. Stroke management was per our stroke care pathway based on the current guidelines for stroke care at our hospital.

Consecutive patients who (a) visited our stroke center within 7 days of an acute ischemic stroke or TIA, and (b) were admitted to our stroke center during the study period and underwent PSG between 2009 and 2016 were considered for eligibility in this study. Prior to enrolling in this study, our neurologist interviewed each patient to assess whether he/she experienced any one of the Obstructive Sleep Apnea (OSA) symptoms such as snoring, observed apnea, or excessive daytime sleepiness. When the patient experienced two or more of the OSA symptoms, polysomnography (PSG) was considered. The exclusion criteria for our study were as follows: (1) age <40 years or more than 85 years; (2) definite history of coronary artery disease; (3) Body Mass Index (BMI) more than 40 kg/m^2^; (4) significant bulbar palsy requiring gastric tube or endotracheal tube insertion; (5) patients who received thrombolytic or endovascular treatments; (6) inadequate period of sleep (<2 h) during the PSG; and (7) refusal to participate in the study. This study was approved by the Severance Hospital Institutional Review Board. The requirement for informed consent was waived because this study was retrospective design and personal information was not used.

### Coronary Artery Computed Tomography

Since July 2006, our stroke center has been using multi-detector coronary computed tomography (MDCT) for detecting hidden asymptomatic CAOD in patients with stroke (10). Briefly, MDCT was consecutively performed when a patient had at least one of the following: (1) presence of atherosclerosis in an intracranial or extracranial cerebral artery; (2) presence of ≥2 risk factors for coronary artery disease such as hypertension, diabetes mellitus, dyslipidemia, cigarette smoking, and central obesity; and (3) old age (males: >45 years, females: >55 years). MDCT was not performed if patients had (1) known CAOD; (2) high pulse rates that were not controlled with beta-blockers at the time of MDCT; (3) poor cooperation; (4) impaired renal function; or (5) failure to obtain informed consent from the patient.

In order to measure the CAC score, scanning was performed with a dual-source CT scanner (Somatom Definition Flash; Siemens Medical Solutions, Erlangen, Germany) using the following parameters: prospective electrocardiographic gating, 120 kVp, 50 mA; field-of-view, 18–20 cm; 0.33 s per rotation. The CAC score (Agatston score) was calculated as the sum of areas in the coronary artery with attenuation exceeding a threshold of 130 HU with score weighting according to the HU (11). In addition, the CAC volumes and CAC mass were measured in the coronary arteries (12). CT coronary angiography was performed after the administration of a non-ionic contrast agent (Iopamiro) with retrospective electrocardiographic gating. Stenosis was considered to be significant when there was more than 50% narrowing in each of the four coronary arteries. The CAC and the presence of significant stenosis in the coronary arteries were reviewed by two cardiac radiologists.

### Polysomnography and Sleep Pattern Assessment

In-laboratory overnight sleep recording (Grass Technologies, Twin PSG Software) was performed. The participants were instructed to go to sleep in a dimly lit, temperature- and noise-controlled sleep monitoring unit. The PSG recording included electroencephalography using frontal, central, and occipital electrodes; 1-lead electrocardiography; electromyography on extraocular eye movement, chin, and bilateral anterior tibialis muscles; nasal airflow and thermistor; peripheral oxygen saturation; sleep position; and chest and abdominal plethysmography. Sleep staging and respiratory- and movement-scoring were done according to the American Academy of Sleep Medicine manual (version 2.0) by a sleep technician with 10 years of experience (13). Based on the respiratory disturbance index (RDI), we classified patients with RDI values between 5/h and 30/h into the mild-to-moderate range for sleep apnea, and patients with RDI values >30/h in the severe range for sleep apnea. The oxygen desaturation index (ODI) was calculated as the number of ≥4% desaturations divided by the total sleep time (in hours). The median days (range) from stroke to PSG were 10.0 days (0, 676).

Each subject was asked the questionnaires including the Pittsburgh Sleep Quality Index (PSQI) (14) Beck Depression Inventory (BDI) (15) Epworth Sleepiness Scale (ESS), and The Snoring, Tiredness, Observed apnea, high blood Pressure-Body mass index, Age, Neck circumference, and Gender (STOP-BANG) score (16) to test the patients' sleep habits and sleep-related problems before the PSG.

### Clinical Variables

We collected demographic information and data such as body mass index and presence of vascular risk factors such as hypertension, diabetes, dyslipidemia, and smoking habits. Underlying cardiovascular diseases and concurrent use of medications including hypnotics were also noted. Data related to lipid profiles were also collected. Stroke severity was assessed using the National Institutes of Health Stroke Scale (NIHSS).

### Statistical Analyses

Statistical analysis was performed using the Windows SPSS package (version 23 IBM Corp., Armonk, NY, USA). Comparisons of clinical variables, PSG findings, and CAC scores between groups were made using the Wilcoxon Mann–Whitney *U*-test for continuous variables and the Chi square test for categorical variables, as appropriate. Spearman's rank correlation was used to identify relationships between CAC measures and the continuous variables. Jonckheere's trend test was performed to compare the CAC scores between groups stratified by the severity of sleep apnea based on the RDI. The effects of sleep-related variables on the CAC score were determined using multiple linear regression analysis while controlling for the effects of age, sex, BMI, and vascular risk factors. The statistical significance threshold was set at two-tailed *p* < 0.05.

## Results

### Baseline Characteristics

During the study period, a total of 32 patients was included in this study. The reason for relatively few patients included in the study is explained in discussion section. Table 1 details the baseline characteristics of the patients recruited in this study. The mean age (±SD) of the study sample was 63.6 (±10.5) years, and 26 (81%) patients were men. Hypertension was the most common (63%, *n* = 20) vascular risk factor, followed by current smoking (28%, *n* = 9), diabetes mellitus (25%, *n* = 8), and hypercholesterolemia (9%, *n* = 3). Six patients reported a clinical history of atrial fibrillation. Univariate analysis of coronary artery calcium scores and clinical and sleep questionnaire variables is shown in Supplementary Table 1.

**Table 1:** Summary of baseline characteristics, polysomnographic findings, and sleep questionnaire scores.

	**All patients** **(*n* = 32)**	**Mild-to-moderate sleep apnea** **(*n* = 9)**	**Severe sleep apnea** **(*n* = 23)**	***p*-value**
**BASELINE CHARACTERISTICS AND VASCULAR RISK FACTORS**				
Age at stroke presentation (years)	64.0 (46–84)	55.0 (46–68)	66.0 (47–84)	**<0.01**
Number of males, *n* (%)	26 (81%)	7 (78%)	19 (83%)	>0.99
Body mass index^§^ (kg/m^2^)	24.4 (18.8–33.0)	22.5 (18.8–33.0)	24.6 (20.4–29.8)	0.08
Smoker, *n* (%)	9 (28%)	4 (44%)	5 (22%)	0.22
Diabetes Mellitus, *n* (%)	8 (25%)	1 (11%)	7 (30%)	0.38
Hypertension, *n* (%)	20 (63%)	4 (44%)	16 (70%)	0.24
Atrial fibrillation, *n* (%)	6 (19%)	1 (11%)	5 (22%)	0.64
Total cholesterol, mmol/L	4.4 (2.6–6.5)	4.9 (3.3–6.5)	3.9 (2.6–6.2)	**0.04**
Low density lipoprotein cholesterol, mmol/L	2.7 (1.2–4.7)	3.1 (1.8–4.0)	2.4 (1.2–4.7)	0.21
Triglyceride, mmol/L	1.2 (0.7–2.5)	1.2 (0.7–2.1)	1.1 (0.7–2.5)	0.93
High density lipoprotein cholesterol, mmol/L	1.1 (0.6–1.7)	1.2 (0.8–1.7)	1.0 (0.6–1.5)	0.38
NIHSS at stroke presentation	1.5 (0–16)	2.0 (0–6)	0 (0–16)	0.96
**POLYSOMNOGRAPHY VARIABLES**				
Sleep efficiency, %	72.1 (24.4–92.4)	80.0 (64.0–92.4)	68.2 (24.4–91.3)	0.12
Sleep stage N1, %	41.8 (9.6–90.0)	25.7 (9.9–90.0)	42.6 (18.7–70.0)	0.08
Sleep stage N2, %	42.9 (10.1–71.5)	54.6 (10.1–71.5)	42.4 (16.6–64.3)	0.12
Sleep stage N3, %	0.0 (0.0–10.6)	0.0 (0.0–10.6)	0.0 (1.1)	0.20
Sleep stage R, %	13.3 (0–35.7)	13.9 (0.0–26.1)	12.2 (0.0–35.7)	0.90
Arousal index (events/hour)	53.0 (17.2–108.0)	35.0 (17.2–75.4)	62.8 (19.8–108.0)	**0.02**
PLMI (events/hour)	8.0 (0.0–96.2)	16.0 (0.0–58.7)	5.5 (0–96.2)	0.13
RDI (events/hour)	44.3 (5.1–109.2)	14.9 (5.1–28.0)	54.6 (31.2–109.2)	**<0.01**
Apnea index (events/hour)	5.9 (0.0–62.7)	0 (0.0–5.3)	21.2 (0.0–62.7)	**<0.01**
O_2_ desaturation index (events/hour)	17.7 (0.0–60.0)	6.0 (0.0–27.0)	35.5 (0.0–60.0)	**<0.01**
Minimum O_2_ saturation (%)	81.2 (66.0–94.0)	86.8 (66.0–94.0)	79.0 (66.0– 88.0)	0.06
Average O_2_ saturation (%)	94.8 (87.9–97.5)	95.9 (93.4–97.5)	94.2 (87.9–95.9)	**<0.01**
**SLEEP QUESTIONNAIRE VARIABLES**				
PSQI	7.0 (1–13)	7.0 (1–10)	7.0 (2–13)	0.86
BDI	10.0 (0.0–23.0)	10.0 (3.0–21.0)	9.5 (0.0–23.0)	0.83
ESS	8.0 (0.0–17.0)	4.0 (0.0–17.0)	9.0 (0.0–17.0)	0.11
STOP-BANG	5.0 (2.0–7.0)	6.0 (2.0–6.0)	5.0 (2.0–7.0)	0.93
**Presence of any CAOD (≥1 vessel)**	13 (41%)	3 (33%)	10 (44%)	0.70

*Data are presented as median (range) or number (percent). PLMI, Periodic limb movement index; RDI, Respiratory Disturbance Index; NIHSS, National Institutes of Health Stroke Scale; PSQI, Pittsburgh Sleep Quality Index; BDI, Beck Depression Inventory; ESS, Epworth Sleepiness Scale; STOP-BANG, The Snoring, Tiredness, Observed apnea, high blood Pressure-Body mass index, Age, Neck circumference, and Gender; CAOD, Coronary Artery Occlusive Disease. P < 0.05 was highlighted with bold characteristics*.

### PSG-Derived Parameters

All the patients had sleep apnea of some degree (mean RDI: 46.3/h ± 25.5/h; mild-to-moderate, *n* = 9; severe, *n* = 23). Thirty (94%) patients had OSA based on the criteria listed in the International Classification of Sleep Disorders, third edition. Two patients were diagnosed with central sleep apnea (central apnea index > 5/h and 50% of total RDI): one patient had an overall RDI of 34.2/h (Cheyne-Stokes breathing and concomitant congestive heart failure), while the other had an RDI of 47.1/h (central sleep apnea syndrome after lateral medullary infarction). The apnea index, ODI, and STOP-BANG score were significantly higher in patients with severe sleep apnea than in patients with mild-to-moderate sleep apnea, while average O_2_ saturation was significantly lower in the former (Table 1). Arousal index was higher in severe OSA group, while periodic limb movement index (PLMI) was not different between the groups.

### The Burden of CAC and Sleep-Disordered Breathing

**Figure 1:**
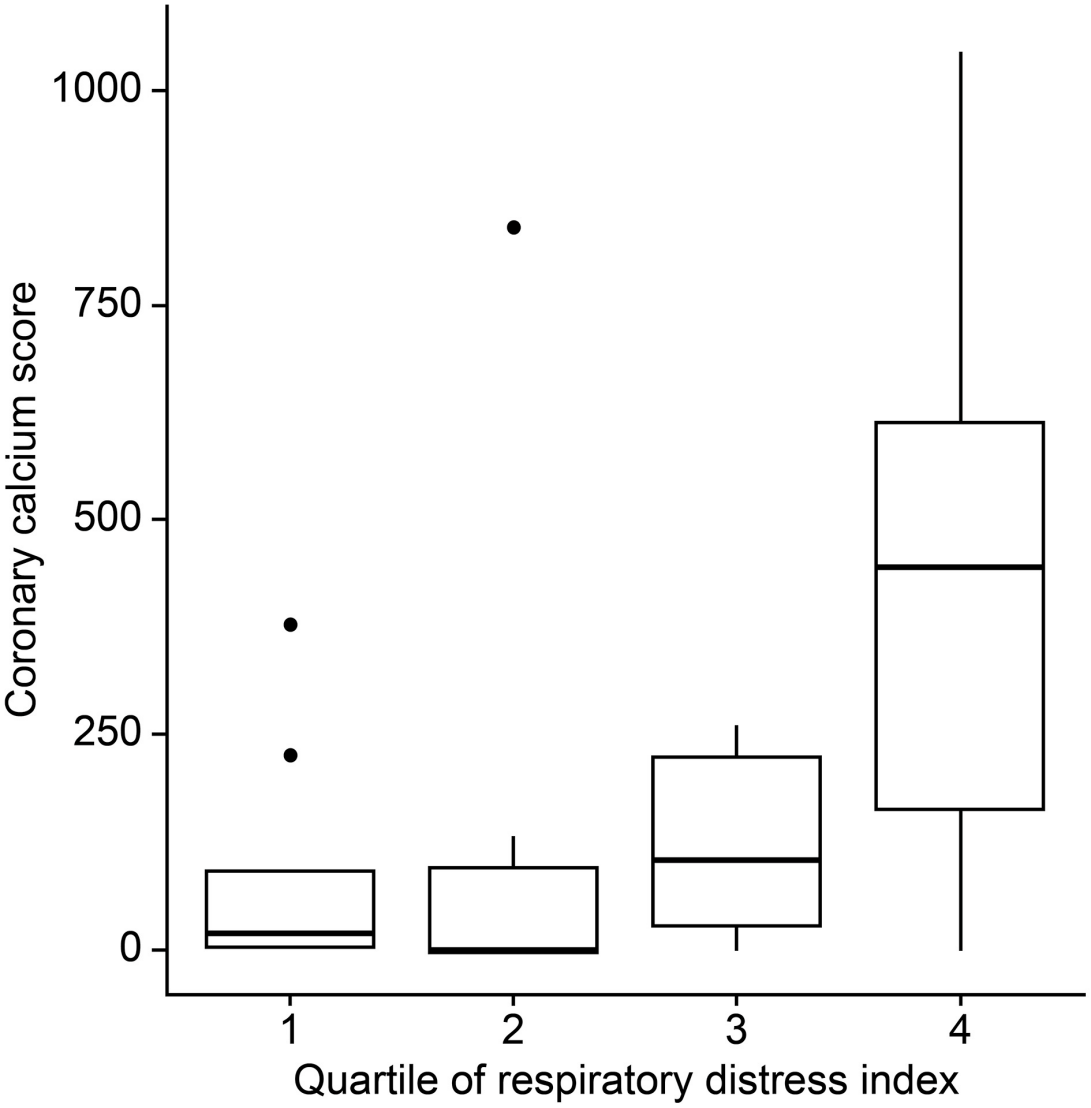
Differences in coronary artery calcium scores by the respiratory disturbance index quartiles.

**Figure 2:**
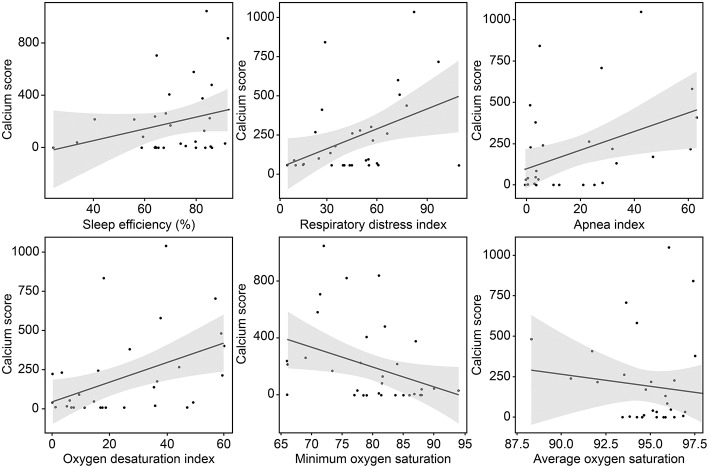
Correlation between the coronary calcium score and variables related to sleep-disordered breathing.

The mean (±SD) CAC score, CAC volume, and CAC mass were 193.0 ± 274.1, 132.3 ± 194.5, and 25.5 ± 35.6, respectively. Twenty-three (72%) patients had a positive CAC score. When we categorized the study population into four groups based on the quartiles of the RDI value, the CAC score was observed to be higher in patients with higher RDI values (Figure 1). Higher CAC scores were associated with elevated RDI value, apnea index, ODI, and STOP-BANG score. Arousal index and PLMI had no significant correlation with CAC. On the other hand, the minimum oxygen saturation was inversely correlated with the CAC score (Figure 2 and Table 2). Linear regression analysis adjusting for the potential determinants of the CAC score such as age, sex, body mass index, total cholesterol, hypertension, diabetes mellitus, and smoking status revealed that the atherosclerotic burden as measured by the CAC score was independently associated with the RDI value (ß [SE] = 5.3 [2.1], *p* = 0.01), ODI (ß [SE] = 6.8 [2.8], *p* = 0.02), and STOP-BANG score (ß [SE] = 90.3 [37.7], *p* = 0.02), while there was no statistically significant association between the CAC score and the apnea index or the minimal and average O_2_ saturation levels (Table 2).

**Table 2:** Multivariate analysis for determinants of the coronary artery calcium scores.

	**ß (SE)**	P^*^
Sleep efficiency	4.1 (3.8)	0.29
Arousal index	3.0 (2.9)	0.31
RDI	5.3 (2.1)	**0.01**
Apnea index	5.3 (3.0)	0.08
O_2_ desaturation index	6.8 (2.8)	**0.02**
Minimum O_2_ saturation	−16.1 (8.3)	0.06
Average O_2_ saturation	−20.3 (33.8)	0.55
PSQI	28.0 (16.2)	0.09
BDI	3.2 (7.5)	0.67
ESS	−4.3 (13.0)	0.74
STOP-BANG	90.3 (37.7)	**0.02**

* *Adjusted for age, sex, body mass index, total cholesterol, hypertension, diabetes mellitus, and smoking status. RDI, Respiratory Disturbance Index; PSQI, Pittsburgh Sleep Quality Index; BDI, Beck Depression Inventory; ESS, Epworth Sleepiness Scale; STOP-BANG, The Snoring, Tiredness, Observed apnea, high blood Pressure-Body mass index, Age, Neck circumference, and Gender. P < 0.05 was highlighted with bold characteristics*.

Thirteen patients showed significant CAOD on ≥1 artery (one vessel, *n* = 7; two vessels, *n* = 5; three vessels, *n* = 1) as revealed by the MDCT. When the study population was classified into two groups, namely patients with significant CAOD (*n* = 13) and patients without significant CAOD (*n* = 19), no group differences were observed in the baseline characteristics. However, the total apnea index (any CAOD, 28.9 ± 24.0; no CAOD, 8.7 ± 10.4; *p* = 0.01) and STOP-BANG score (any CAOD, 5.9 ± 1.0; no CAOD, 4.3 ± 1.5; *p* = 0.001) were higher in patients with significant CAOD than in patients without significant CAOD.

### Treatment

According to management guideline of OSA, we recommended to all of subjects to use CPAP (continuous positive airway pressure, including CSB and CSA patients, after in-lab pressure titration), or APAP (automatic positive airway pressure) therapy. As results, only 7 (22%) of OSA started receiving treatment. The reason for not getting PAP treatment after PSG was as follows; poor cooperation (*n* = 12), economic issues (*n* = 11), and need for active rehabilitation of stroke sequalae (*n* = 2). Among 7 subjects who started PAP treatment, 4 are still using PAP therapy with good compliance (follow-up years >2).

## Discussion

While the severity of sleep-disordered breathing has been generally known to be correlated with systemic atherosclerosis including CAOD, the significance of sleep apnea in patients with stroke who are at high risk for future cardiovascular events has not been duly recognized, given the lack of direct evidence supporting it. Our findings indicate a strong association between the severity of sleep apnea and the atherosclerotic burden as measured by the CAC score. Furthermore, this association remained significant after controlling for the effects of age, sex, BMI, and other vascular risk factors.

There were two studies evaluating relationship between CAC and OSA, in which subjects are people with non-symptomatic CAD (17, 18). They commonly found correlation of OSA severity and CAC. However, there are a few differences between our finding and them. The work done in 2008 (17) set major dependent variable as presence of CAC (binary variable, CAC > 0 or not). This is because subjects having severe degree of CAC were very few. In this study, even fourth high quartile AHI (apnea-hyponea index) group had median of 44 agatson score, which is much less than our result. Even more, in this study, CAC did not correlate with O2 nadir. Instead of explaining it with intermittent hypoxia which is generally accepted core pathomechanism of atherosclerosis, they presumed that stressors such as sleep time and frequent arousals might effect on CAC in their subjects.

The other recent research paper (18) recruiting subjects having health screenings, revealed only O2 nadir was independently associated with CAC score. Although O2 nadir had trend for inverse relationship with CAC in our study, the factors having independent association were RDI and ODI. We assume that not O2 level itself, but frequency of desaturation event has much stronger factor for CAC, in our population. Moreover, we also used structured questionnaire, consisting of BDI, PSQI, ESS, and STOP-BANG. Among them, STOP-BANG scale was independently associated with CAC after adjustment for conventional vascular risk factors.

Detection of underlying CAD is essential because CAOD, asymptomatic or symptomatic, is a strong predictor of future cardiovascular events in patients with stroke (19). Previously, the potential candidate for further CAD evaluation was primarily based on the traditionally known vascular risk factors such as hypertension, diabetes mellitus, dyslipidemia, or cigarette smoking, or the presence of concomitant cerebral atherosclerosis (20, 21). Sleep apnea, a potential risk factor for CAD, could be used for predicting the presence of hidden CAD. However, previous studies have not investigated the relationship between the CAC score and OSA (7, 8, 22). One study with a large sample size examined the relationship between OSA and CAC in otherwise healthy subjects, including individuals with obesity (23). However, Korean patients with OSA or stroke are generally not obese and that study did not evaluate patients with stroke. In one study with the largest number of subjects, the presence of OSA was based only on clinical symptoms (24) and not PSG findings. In this study, we demonstrated an association between the atherosclerotic burden measured by the CAC score and the severity of OSA confirmed by PSG findings. Our results emphasize the utility of PSG in determining the presence and severity of sleep apnea and using it to identify hidden CAD among patients with ischemic stroke.

Multiple pathophysiological mechanisms underlying the relationship between sleep apnea and the development of cardiovascular and cerebrovascular diseases have been proposed: intermittent hypoxia, sympathetic activation, or inflammation (25–27). Intermittent hypoxia, represented by RDI and ODI in sleep apnea, is known to accentuate free radical production and lipid oxidation (28, 29). A previous study showed that the level of C-reactive protein, a reliable inflammatory marker, was significantly higher in patients with OSA than in control subjects (30). These detrimental effects of sleep apnea could result in elevated atherosclerotic burden in patients with stroke with more severe sleep apnea, as shown in this study.

Our study also found that the STOP-BANG score, primarily a screening tool for diagnosing OSA, is independently associated with the CAC score. This suggests that the use of a simple questionnaire might be beneficial in the screening of asymptomatic CAOD in patients with ischemic stroke. Although we did not evaluate the relationship between biomarkers and sleep apnea, the association between oxygen desaturation, ODI, and CAC demonstrated in our study represents adverse biological consequences of intermittent hypoxia on coronary artery health.

Recent large-scale trial examining the effectiveness of CPAP therapy failed to prevent secondary coronary events in patients with already established coronary artery disease (31), which emphasize “primary” prevention of coronary events with CPAP treatment. Also, the complex pathophysiology of OSA suggests that new therapies or combination therapy might be more effective than a CPAP therapy alone (32, 33).

Contrary to the previous findings (31, 34, 35) ESS was quite low in this study. This may be explained in a few ways. In our study population, Patients might had high level of anxiety for stroke progression (in acute stage) and recurrence (in chronic stage), combined unfamiliar situation with attached PSG device, leading to “hyperarousal state” (36). BDI, used in our study, has known to have significant relationship and overlap with anxiety scales (37). It is also known that a person who has 13 or more BDI score are at risk for developing mood problem. In our study, subjects with BDI>13 were ~33%. Therefore, it might be postulated that the patient with mood problem might have reported low daytime sleepiness. Another previous study suggested that there may be a relationship between lower ESS and the severity of disability and the ESS is not a sensitive marker of hypersomnolence or OSA in stroke patients (38). Most of physical needs in stroke patients with disability are met by hospital personnel or caregiver might result in less feeling subjective sleepiness in these patients (38). In addition, the use of drug having potential central stimulating effects such as beta-blockers, statins, antithrombotics could the affect patients' sleepiness level (39).

Recently published data showed short (<6 h) sleep duration and frequent arousal is related with carotid and coronary atherosclerosis (40, 41). However, we did not observe these correlations. Possible cause is that most of our patients had short total sleep time and high arousal index during PSG (median and interquartile range of sleep time, 4.7 and 0.9 h; arousal index, 53.9/h, 35.7, respectively). The two factors possibly aggravated CAC, but both small sample size and relatively homogenous characteristics (most were bad sleepers) might make the statistical power decrease. Although not reached the statistical significance (*p* = 0.21), another counterintuitive finding is that higher sleep efficiency seemed to be related with higher CAC (Figure 1). Similarly, the low sleep efficiency might be related with shorter total sleep time in which respiratory events are recorded sufficiently.

Interestingly, the apnea index value was not found to be significantly associated with the CAC score in this analysis. This finding was consistent even after removal of two CSA patients. The lack of association between apnea index and CAC might be ascribed to the fact that apnea events *per se* are far less frequent than hypopnea and respiratory effort-related arousals. The overall degree of oxygen desaturation and its frequency showed stronger effects on the CAC score than apnea alone. We did not find any significant difference in the values of the vascular risk factors between groups assigned according to the CAC score, although the proportion of each vascular risk factor tended to be higher in patients with higher RDI values. Further, we found that the sleep apnea-derived measures that could be confirmed by the PSG study were significantly associated with the CAD burden. This finding points to the superior potential of PSG in identifying hidden CAD in patients with ischemic stroke compared to that of conventional vascular risk factors (24).

The rate of receiving CPAP treatment was quite low. After acute stage, the stroke patients usually were followed up by stroke specialist, not a physician who cares sleep disorder. Therefore, it is necessary to force them to visit regular to recommend treatment repeatedly, even after initial denial for receiving treatment.

The limitations of the current study must be addressed. First, patients with high risk for OSA (having snoring, observed apnea, or daytime sleepiness) and relatively mild stroke symptoms were recruited in the study. Second, the sample size of our study was not large enough for us to generalize our findings to the wider population of patients with stroke. We also thought one of limitations was low number of patients included in this study. This was caused by patient's refusal or reluctance to PSG due to uncomfortable device, or unstable neurologic status within acute stroke period, along with lack of personnel or PSG room availability. Actually, in previous studies of PSG for acute stroke patients, the sample size was not large, which might be related with some difficulties we also experienced. Furthermore, we could not perform the MDCT for detecting hidden asymptomatic CAOD coronary because of change in Korean government's insurance issue in a significant number of cases. These led in small number of patients included in this study. Finally, long-term prognosis including parameters such as number of future cardiovascular events was not analyzed as a part of this study. A follow-up study may be required to clarify the significance of OSA in the presentation of cardiovascular outcomes.

## Conclusions

Patients with ischemic stroke show significant sleep-disordered breathing, such that higher severity of breathing distress is associated with higher silent coronary artery atherosclerosis burden. Given the relationship between sleep apnea and coronary atherosclerosis, proper intervention and management of sleep apnea might improve mortality outcomes among patients with ischemic stroke.

## Data Availability

The datasets for this manuscript are not publicly available because of protection for personal information. Requests to access the datasets should be directed to YK, neuro05@yuhs.ac.

## Ethics Statement

This study was approved by the Severance Hospital Institutional Review Board.

## Author Contributions

KC: acquisition of data, analysis and interpretation of data, writing of original draft. DL, KK, and YC: acquisition of data and interpretation of data. HN, JH, and KH: interpretation of data and critical revision of the manuscript for intellectual content. YK: study concept and design, analysis and interpretation of data, critical revision of the manuscript for intellectual content.

### Conflict of Interest Statement

The authors declare that the research was conducted in the absence of any commercial or financial relationships that could be construed as a potential conflict of interest.
