# Supplementary material for: Relationship Between Sleep Apnea and Coronary Artery Calcium in Patients With Ischemic Stroke
Source: Front Neurol. 2019 Jul 31;10:819. doi: 10.3389/fneur.2019.00819 (PMC6684953; doi:10.3389/fneur.2019.00819)
Supplement: Supplementary file 1 [file Table_1.DOCX]

Supplementary Material

**Supplementary Table 1.** Univariate analysis of coronary artery calcium scores and clinical and sleep questionnaire variables.

|  | ß (SE) | *P* value |
| --- | --- | --- |
| **Vascular risk factors** | | |
| Age | 0.87 (4.77) | 0.85 |
| Sex | -3.27 (126.20) | 0.98 |
| Body mass index | -2.60 (16.69) | 0.87 |
| Smoking | 32.04 (109.4) | 0.77 |
| Diabetes Mellitus | 119.57 (111.64) | 0.29 |
| Hypertension | 104.20 (99.95) | 0.30 |
| Atrial fibrillation | 182.08 (121.75) | 0.14 |
| Total cholesterol | 8.2 (46.1) | 0.86 |
| Low density lipoprotein cholesterol | 10.5 (53.1) | 0.85 |
| Triglyceride | -19.6 (113.8) | 0.86 |
| High density lipoprotein cholesterol | 121.1(175.0) | 0.49 |
| **Polysomnography related variables** | | |
| Sleep efficiency | -124.2 (218.0) | 0.57 |
| RDI | - 1. (1.8) | 0.01 |
| Apnea index | - 1. (2.3) | 0.01 |
| O_2_ desaturation index | - 1. (2.3) | <0.01 |
| Minimum O_2_ saturation | -13.9 (6.2) | 0.03 |
| Average O_2_ saturation | -15.2 (24.4) | 0.53 |
| **Sleep questionnaire variables** | | |
| PSQI | 27.6 (14.6) | 0.07 |
| BDI | 5.8 (6.4) | 0.37 |
| ESS | -1.9 ((11.2) | 0.86 |
| STOP-BANG | 91.7 (28.5) | <0.01 |

RDI, Respiratory Disturbance Index; NIHSS, National Institutes of Health Stroke Scale; PSQI, Pittsburgh Sleep Quality Index; BDI, Beck Depression Inventory; ESS, Epworth Sleepiness Scale; STOP-BANG, The Snoring, Tiredness, Observed apnea, high blood Pressure-Body mass index, Age, Neck circumference, and Gender; CAOD, Coronary Artery Occlusive Disease.
